# Characterization of a Galactose Oxidase from *Fusarium odoratissimum* and Its Application in the Modification of Agarose

**DOI:** 10.3390/foods12030603

**Published:** 2023-02-01

**Authors:** Na Cao, Guangli Xia, Huihui Sun, Ling Zhao, Rong Cao, Hong Jiang, Xiangzhao Mao, Qi Liu

**Affiliations:** 1 College of Food Science and Engineering, Ocean University of China, Qingdao 266003, China; 2 Department of Food Engineering and Nutrition, Yellow Sea Fisheries Research Institute, Chinese Academy of Fishery Sciences, Qingdao 266071, China; 3 Laboratory for Marine Drugs and Bioproducts of Qingdao National Laboratory for Marine Science and Technology, Qingdao 266237, China

**Keywords:** galactose oxidase, *Fusarium odoratissimum*, enzyme characterization, oxidation modification, polyaldehyde-based polymer, agarose

## Abstract

A galactose oxidase gene, *gao-5f*, was cloned from *Fusarium odoratissimum* and successfully expressed in *E. coli*. The galactose oxidase GAO-5F belongs to the AA5 family and consists of 681 amino acids, with an estimated molecular weight of 72 kDa. GAO-5F exhibited maximum activity at 40 °C and pH 7.0 and showed no change in activity after 24 h incubation at 30 °C. Moreover, GAO-5F exhibited 40% of its maximum activity after 24 h incubation at 50 °C and 60% after 40 h incubation at pH 7.0. The measured thermostability of GAO-5F is superior to galactose oxidase’s reported thermostability. The enzyme exhibited strict substrate specificity toward D-galactose and oligosaccharides/polysaccharides containing D-galactose. Further analysis demonstrated that GAO-5F specifically oxidized agarose to a polyaldehyde-based polymer, which could be used as a polyaldehyde to crosslink with gelatin to form edible packaging films. To our knowledge, this is the first report about the modification of agarose by galactose oxidase, and this result has laid a foundation for the further development of edible membranes using agarose.

## 1. Introduction

Renewable resources are economical, green, abundant, and degradable, and are essential in developing the food industry, and packaging, biomedicine, and other fields [1]. The development of green and environmentally friendly packaging materials using renewable resources is of great significance to solve the environmental problems caused by petroleum-based plastics and to improve the quality of food [2]. Edible packaging film prepared from natural edible materials comes into being and has promising application prospects in the food industry [3,4]. Among them, edible film made from gelatin as the main raw material has the advantages of being green, non-toxic, non-hazardous, and biodegradable. Therefore, gelatin edible film is a new packaging material with great potential to replace petroleum-based plastic packaging materials [5]. However, the mechanical strength, flexibility, and hydrophobicity of single gelatin edible film have certain defects, which represent a bottleneck restricting its industrial production and commercial application, so there is an urgent need to improve the performance of gelatin edible film. One of the effective ways to improve the performance of gelatin edible films is to use an aldehyde-containing crosslinking agent for crosslinking modification, which can enhance the stability of the film structure and improve its overall performance [6,7]. By introducing crosslinking agents, a new three-dimensional grid structure can be formed within/between gelatin molecules, which can enhance the stability of the film structure and improve the overall performance of the film. In recent years, many scholars have tried to use the oxidized products of natural polysaccharides, such as oxidized starch [8] and oxidized alginate [9], in the crosslinking modification of gelatin film, and have gradually made some progress in improving the performance of edible gelatin film. Therefore, this is a potential way to modify gelatin by using crosslinking agents with oxidized polysaccharides.

Among natural polymers from terrestrial and marine sources, polysaccharides with good biological properties have received widespread attention [10,11]. Marine polysaccharides are renewable natural resources that are abundant and inexpensive [12,13]. Studies have shown that agarose, compared with other marine polysaccharides, has good renewability, biodegradability, water resistance, moisturizing capacity, edibility, mechanical properties, and unique gel-sol transition characteristics, and can form edible packaging films with suitable properties as an alternative material to traditional food packaging [14,15]. Since polysaccharides are missing reducing carbonyl groups, modification is required for further crosslinking [16]. Commonly, 2,2,6,6-tetramethylpiperidine-1-oxyl (TEMPO) could catalyze the oxidation of primary hydroxyls in polysaccharides selectively and efficiently to aldehyde groups under mild conditions [17], but excess oxidants could further convert some aldehyde groups to carboxyl groups [18]. Compared to chemical catalysis, it is more advantageous to produce them with galactose oxidase, which could oxidize a variety of primary alcohols, especially galactose with a C6 hydroxyl group, to produce the corresponding aldehydes while reducing oxygen to hydrogen peroxide in the catalytic reaction [19]. Agarose molecules are abundant in C6 primary hydroxyl functional groups, which can be converted into polyaldehyde-based polymers by specific oxidation with galactose oxidases. The multi-aldehyde polymers can further crosslink with gelatin to obtain edible packaging films that meet practical applications in the food industry.

Galactose oxidase (E.C. 1.1.3.9) is a monomeric free radical copper oxidase with copper ions as a cofactor [20]. This extracellular monomeric enzyme is secreted by some filamentous fungi, mainly *Fusarium* species [21]. Galactose oxidases are classified in the Carbohydrate Active Enzyme Database as members of the newly established auxiliary active (AA) family AA5 [22]. This family includes copper radical oxidases and two subfamilies, AA5_1 and AA5_2, containing glyoxal oxidase and galactose oxidase, respectively, which have similar three-dimensional structures and active sites but different catalytic properties [23]. Galactose oxidase has strict regioselectivity for the substrate galactose, oxidizing only the hydroxyl group at the C6 position and strictly differentiating between galactose and glucose, and glucose cannot be used as a substrate by galactose oxidase [24]. However, galactose oxidase has a broad spectrum of substrates and can oxidize monosaccharides, polysaccharides, aliphatic and aromatic alcohols, polyols, and many other compounds containing galactose units [25]. Some studies have been reported about galactose oxidase-catalyzed oxidation of galactose-containing polysaccharides, including spruce galactoglucomannan, guar galactomannan, larch arabinogalactan, corn fiber arabinoxylan, and tamarind seed xyloglucan [26]. These characteristics enable the use of galactose oxidase in various fields, such as biosensors, chemical synthesis, biomedicine, and molecular modifications [27,28]. In biosensors, this enzyme can be used for the quantitative detection of galactose, including the detection of lactose content in dairy products and the timely detection of lactose content in blood for galactosemia [29,30]. In synthetic applications, a one-pot tandem enzyme reaction using galactose oxidase and aldehyde oxidase was used to convert hydroxymethylfurfural to the pure bioplastics precursor furandicarboxylic acid [31]. Galactose oxidases from *F. sambucinum* and *F. oxysporum* have been successfully cloned and expressed in *E. coli* and yeast [32,33].

In this paper, we report the heterologous expression of a galactose oxidase from *F. odoratissimum* in *E. coli*. The purified recombinant galactose oxidase GAO-5F was biochemically characterized. The most commonly used method for galactose oxidase determination is to monitor the production of H_2_O_2_, using 2,2′-Azino-bis (3-ethylbenzthiazoline-6-sulfonic acid) (ABTS) or o-dianisidine [25,34]. Considering the toxicity of these, ABTS was employed in characterizing galactose oxidase. In addition, for the first time, the galactose oxidase GAO-5F was used for oxidation of agarose to yield a polyaldehyde-based polymer, which can be further used to crosslink with gelatin to produce edible packaging films.

## 2. Materials and Methods

### 2.1. Schematic Overview of the Experimental Program

**Figure 1:**
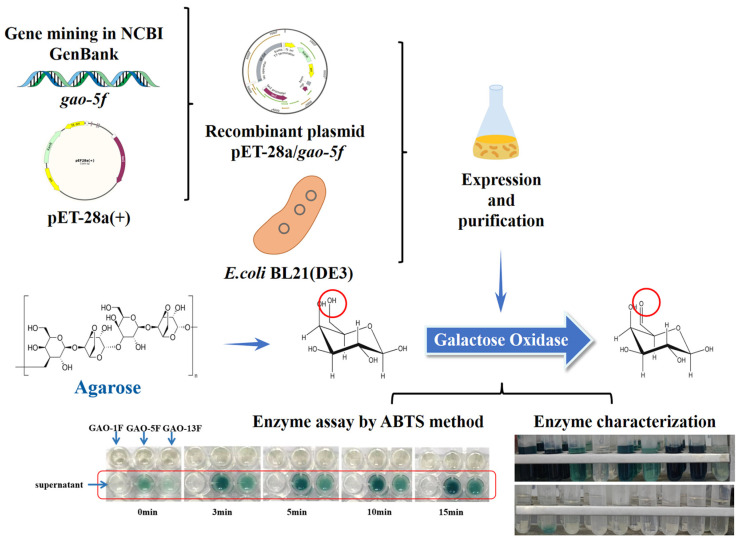
The schematic overview of the experimental program. The galactose oxidase mined from NCBI GenBank was expressed, purified and characterized, and its applicaiton in the modification of agarose.

In this study, novel galactose oxidases were mined from NCBI GeneBank. The target gene was expressed in *E. coli* successfully. The purified enzyme was characterized for its biochemical properties. Following up on its oxidation properties, the polyaldehyde-based polymers were prepared using galactose oxidase-catalyzed oxidation with agarose as the substrate. The schematic overview of the experimental program is shown in Figure 1.

### 2.2. Materials, Strains and Culture Conditions

D-galactose was purchased from Sinopharm Chemical Reagent Co., Ltd. (Beijing, China). ABTS and horseradish peroxidase were purchased from MACKLIN (Shanghai, China). All other chemicals and reagents used were of analytical grade and were purchased from local markets. SDS-PAGE protein standard was from BioRad (Herts, UK). *E. coli* BL21 Star™ (DE3) strains (Tiangen Biotech, Beijing, China) were used as the prokaryotic expression host.

### 2.3. Gene Mining and Sequence Analysis

Searching for novel galactose oxidase gene sequences was performed using the BLASTP program (https://blast.ncbi.nlm.nih.gov/Blast.cgi?PROGRAM=blastp&PAGE_TYPE=BlastSearch&BLAST_SPEC=&LINK_LOC=blasttab&LAST_PAGE=blastx, accessed on 18 January 2023) in the NCBI database. The galactose oxidase genes *gao-1f* from *F. oxysporum* (GenBank: KB729968.1), *gao-5f* from *F. odoratissimum* (GenBank: XM_031208735.1) and *gao-13f* from *F. flagelliforme* (GenBank: PXXK01000252.1) were selected. Signal peptide was analyzed at SignalP 6.0 server (https://services.healthtech.dtu.dk/service.php?SignalP, accessed on 18 January 2023). Sequence alignments were conducted using Clustal W program [35] and ESPript 3.0 (https://espript.ibcp.fr/ESPript/ESPript/, accessed on 18 January 2023). Phylogenetic relationships among the galactose oxidase were analyzed through a neighbor-joining method packaged in MEGA 6.06 [36].

### 2.4. Expression of the Three Galactose Oxidase Genes in E. coli

To explore their potential catalytic performance, the above three optimized codon genes, *gao-1f*, *gao-5f,* and *gao-13f*, encoding the galactose oxidases GAO-1F, GAO-5F, and GAO-13F without signal peptides were synthesized by Sangon Biotech (Shanghai, China). The genes were ligated to the pET-28a(+) vector, respectively. The resulting plasmids pET-28a-GAO-1F, pET-28a-GAO-5F, and pET-28a-GAO-13F were then transformed into *E. coli* BL21 (DE3) competent cells for expression of the galactose oxidases. The constructed plasmids and their corresponding recombinant strains were stored at −80 °C. Recombinant *E. coli* BL21 (DE3) cells harboring galactose oxidase genes in the pET-28a(+) vector, including GAO-1F, GAO-5F, and GAO-13F, were cultivated in LB medium containing kanamycin (50 μg/mL) at 37 °C for ~3 h with orbital shaking at 200 rpm. When the absorbance at 600 nm of the culture broth reached 0.6, overexpression of protein was induced by adding IPTG at a final concentration of 0.1 mM. The induced cultures were further incubated for 18 h at 25 °C (200 rpm). Then, cells were collected by centrifugation at 4 °C (8000× *g*) for 10 min, washed twice with 0.85% NaCl solution, and stored at −20 °C.

### 2.5. Purification of the Three Galactose Oxidases

The recombinant proteins were purified by nickel affinity chromatography. Firstly, the harvested cells were resuspended in Tris-HCl buffer (50 mM, pH 8.0), then disrupted by ultrasonication. The cell debris was removed by centrifugation (10,000× *g*, 4 °C) for 30 min. The supernatants containing the crude enzymes were filtered through a 0.45 μm filter and then loaded onto a Ni-NTA column (1 mL; Qiagen, Hilden, Germany) at a flow rate of 1 mL/min. The column was preequilibrated with buffer A (50 mM NaH_2_PO_4_, 300 mM NaCl, and 10 mM imidazole, pH 8.0). Then, the column was washed with 5 column volumes of buffer B (50 mM NaH_2_PO_4_, 300 mM NaCl, and 20 mM imidazole, pH 8.0), followed by 5 column volumes of buffer C (50 mM NaH_2_PO_4_, 300 mM NaCl, and 50 mM imidazole, pH 8.0), 10 column volumes of buffer D (50 mM NaH_2_PO_4_, 300 mM NaCl, and 150 mM imidazole, pH 8.0) and 5 column volumes of buffer E (50 mM NaH_2_PO_4_, 300 mM NaCl, and 200 mM imidazole, pH 8.0) at a flow rate of 1 mL/min. The eluted proteins were collected respectively, then desalted with Tris-HCl buffer (50 mM, pH 8.0) and concentrated by ultrafiltration using a 50 mL Amicon Ultra Centrifugal Filter Device with a molecular weight cutoff of 30 kDa (Millipore, Burlington, MA, USA) for subsequent experiments. The supernatants of the cell lysates and purified proteins were analyzed by 12.5% sodium dodecyl sulfate-polyacrylamide gel electrophoresis (SDS-PAGE). Protein concentration was determined using the Bradford Protein Assay Kit purchased from Tiangen Biotech (Beijing, China), using bovine serum albumin as the standard. All purification steps were carried out at 4 °C.

### 2.6. Enzyme Assay

The enzymatic activity of galactose oxidase was determined by the ABTS indirect detection method using a peroxidase coupling assay [37]. Galactose oxidase catalyzes the oxidation of primary alcohols to corresponding aldehydes and reduces oxygen to hydrogen peroxide in the catalytic reaction. ABTS, as a color developing agent, can be oxidized by hydrogen peroxide to produce blue-green cationic free radicals with maximum absorption at 420 nm. The standard assays were performed in a reaction mixture (2.7 mL) containing ABTS (4.41 mg) in phosphate buffer (100 mM, pH 7.0), horseradish peroxidase (45 U), D-galactose (90 mM), NaCl (150 mM), and 10 μL purified galactose oxidase. The mixture was incubated at 40 °C for 3 min and then the absorbance at 420 nm was measured. All the reactions were performed in triplicate and their mean and standard deviation values were used as the final results. One unit (U) of galactose oxidase activity was defined as the amount of enzyme required to oxidize 2 μmol ABTS or 1 μmol D-galactose per minute under the above conditions, which is equal to 1 μmol O_2_ consumed per min.

### 2.7. Enzyme Characterization

D-galactose (90 mM) was used as the substrate in the enzymatic characterization of GAO-5F. The optimal temperature for GAO-5F activity was determined in 20 mM phosphate buffer (pH 7.0) over the temperature range of 20–60 °C. Different buffer solutions, including sodium citrate buffer (20 mM, pH 3.0 to 6.0), phosphate buffer (20 mM, pH 6.0 to 8.0), Tris-HCl buffer (20 mM, pH 8.0 to 9.0), and glycine-NaOH buffer (20 mM, pH 9.0 to 10.0) were used to determine the optimum reaction pH of GAO-5F. The relative activities under optimal temperature and pH conditions were defined as 100%.

The thermal stability of purified GAO-5F was determined to test the residual enzyme activity by incubating the enzyme at 30, 40, and 50 °C for different times. GAO-5F was incubated in phosphate buffer (pH 6.0 to 8.0) at 40 °C for several hours, and then the pH stability was measured in 20 mM phosphate buffer (pH 7.0).

The kinetic parameters of GAO-5F toward D-galactose with different concentrations (30 to 300 mM) were determined. The reaction mixture was incubated at 40 °C for 3 min, and the change in absorbance was detected by a spectrophotometer. *K*_m_ and *V*_max_ of the purified GAO-5F were calculated by nonlinear regression analysis and the Michaelis–Menten equation using Origin 9.0 (OriginLab, Northampton, MA, USA).

The effects of different metal ions and chemicals on recombinant GAO-5F activities were determined by pre-incubating the enzyme in 20 mM phosphate buffer (pH 7.0) with a series of metal ions, including Na^+^, K^+^, Mg^2+^ and Cu^2+^, and chemicals, including EDTA, hexadecyl trimethyl ammonium bromide (CTAB), SDS, and Tween 80. Since Cu^2+^ precipitates in phosphate buffer (pH 7.0) to form insoluble copper phosphate, the phosphate buffer was replaced with ddH_2_O (pH 7.0) in the determination of the effect of Cu^2+^ on the activity of GAO-5F. All compounds except Tween 80 (2.5%) were added to a final concentration of 5 mM. The catalytic activity without added metal ions was used as the control, and the activity was defined as 100%.

The substrate specificity of GAO-5F was determined under standard conditions using different substances, including methyl-α-D-galactoside, D-galactose (relative activity was set at 100%), D-(+)-glucose, sucrose, lactose monohydrate, maltose, D-(+)-melibiose, D-(+)-raffinose, soluble starch, fructooligosaccharides, guar gum, gum arabic, agarose, and *κ*-carrageenan. The final concentration of all substrates was 25 mM, except for soluble starch, fructooligosaccharides, guar gum, gum arabic, agarose, and *κ*-carrageenan, which were used at a concentration of 1% (*w*/*v*).

### 2.8. Oxidation of Agarose by GAO-5F

The reaction mixture was composed of phosphate buffer (20 mM, pH 7.0), 15 mg agarose (boiled for 10 min to dissolve), and 50 U purified enzyme of GAO-5F. The mixture was incubated at 40 °C for 30 min and then kept at 4 °C for 1 h. The unreacted substrate agarose gel was removed by centrifugation at 8000× *g* for 10 min. The reaction product and the substrate were analyzed by Fourier Transform Infrared (FTIR) spectrometry (ThermoScientific Nicolet IS5, Waltham, MA, USA) against the background of a blank KBr pellet. The spectra were produced with a wave number range from 4000 to 400 cm^–1^ and at a resolution of 4 cm^–1^ over 32 cumulative scans.

## 3. Results and Discussion

### 3.1. Sequences Analysis

**Figure 2:**
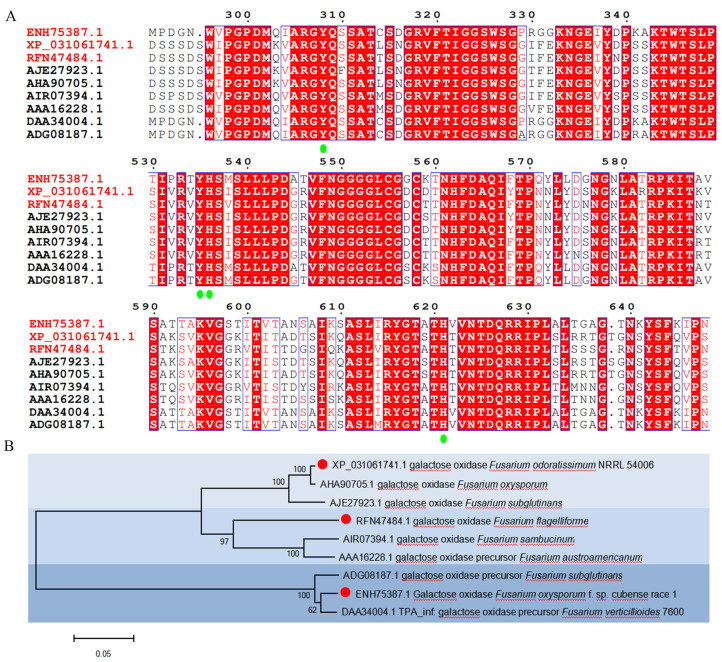
Bioinformatic analysis of galactose oxidases from *Fusarium* species. (**A**) Multiple amino acid sequence alignment of galactose oxidases from *Fusarium* species. The typical catalytic sites (Tyr, His, Tyr, and His) were emphasized with green oval. (**B**) Neighbor-joining tree of galactose oxidases from *Fusarium* species.

Based on the GenBank database mining, three galactose oxidase genes, *gao-1f*, *gao-5f*, and *gao-13f* from *F. oxysporum*, *F. odoratissimum,* and *F. flagelliforme*, respectively, were selected from the NCBI database. The full-length GAO-1F, GAO-5F, and GAO-13F genes had open reading frames of 2040, 2046, and 2043 bp encoding 680, 682, and 681 amino acids, including predicted signal peptides of 20, 26, and 16 amino acids, respectively. All of the three galactose oxidases without signal peptides were synthesized with codon optimization. Multiple sequence alignment with other reported galactose oxidases from *Fusarium* species was conducted, and the results showed that their catalytic domains contained four key active sites (Y308, Y535, H536, and H621), which are conserved in all selected galactose oxidases (Figure 2A) [38]. To gain a better understanding of the relationships involved in the molecular evolution of these galactose oxidases, a phylogenetic tree comprising GAO-1F (ENH75387.1), GAO-5F (XP_031061741.1), and GAO-13F (RFN47484.1) with other reported *Fusarium*-derived galactose oxidases was constructed (Figure 2B). The results showed that most of them exhibited high homology except GAO-1F, DAA34004.1, and ADG08187.1. The catalytic activity of DAA34004.1 and ADG08187.1 has not been reported. The galactose oxidase activities of GaoA (GenBank AJE27923.1) from *F. subglutinans* and GalOx (GenBank AHA90705.1) from *F. oxysporum*, which showed the highest homologous with GAO-5F, were 1255 U/mg (with a production of 1.38 mg/L of culture liquid medium) and 65.4 U/mg (with a production of 5.7 mg/L of soluble protein), respectively [33,34]. GAO-13F showed the highest identities with galactose oxidases GaoA from *F. sambucinum* (AIR07394.1) [32], and GAOX from *F. oxysporum* (AAA16228.1) [39]. The catalytic activity of GaoA was 159 U/mg [32], and the quality and yield of GAOX reached 500 mg/L with optimization of culture conditions [39].

### 3.2. Enzyme Expression, Purification, and Molecular Properties

**Figure 3:**
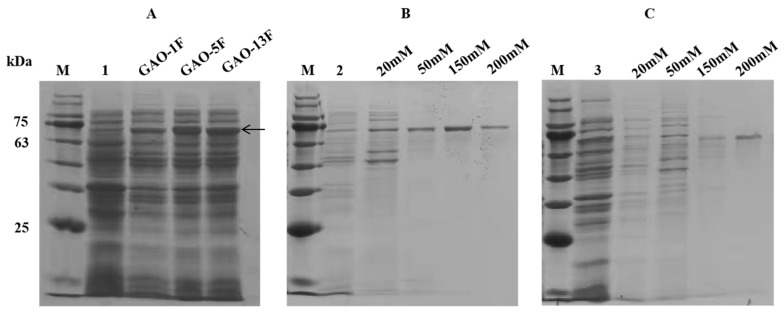
SDS-PAGE analysis of purified GAO-1F, GAO-5F, and GAO-13F. Lane M, protein molecular mass marker. (**A**) Supernatants of GAO-1F, GAO-5F, and GAO-13F cell lysates, respectively. (**B**) Elution of GAO-5F using different concentrations of imidazole. (**C**) Elution of GAO-13F using different concentrations of imidazole. Lane 1, supernatant of *E. coli* BL21(DE3)/pET28a; lane 2, flow-through fluid of GAO-5F; lane 3, flow-through fluid of GAO-13F.

The recombinant plasmids pET-28a-GAO-1F, pET-28a-GAO-5F, and pET-28a-GAO-13F with a 6×His tag at the N-terminus were constructed by gene synthesis, and these plasmids were successfully transformed into *E. coli* BL21(DE3) cells for expression. The three proteins were successfully expressed and purified to electrophoretic homogeneity using a Ni-NTA column. Three single clear bands with a molecular mass of approximately 72 kDa were visible on SDS-PAGE (Figure 3), and were seemingly consistent with those predicted by the deduced amino acid sequences. Most reported galactose oxidases from *Fusarium* species have molecular weights about 70 kDa, such as GalOx from *F. sambucinum* (68.5 kDa) [32], and GalOx from *F. oxysporum* (70 kDa) [33]. The enzyme activities of the purified enzymes were detected, and the specific activities of GAO-5F and GAO-13F were 62.837 U/mg and 38.636 U/mg, respectively, whereas GAO-1F was inactive (Table 1). Thus, GAO-5F was selected for further characterization.

### 3.3. Characterization of GAO-5F

**Figure 4:**
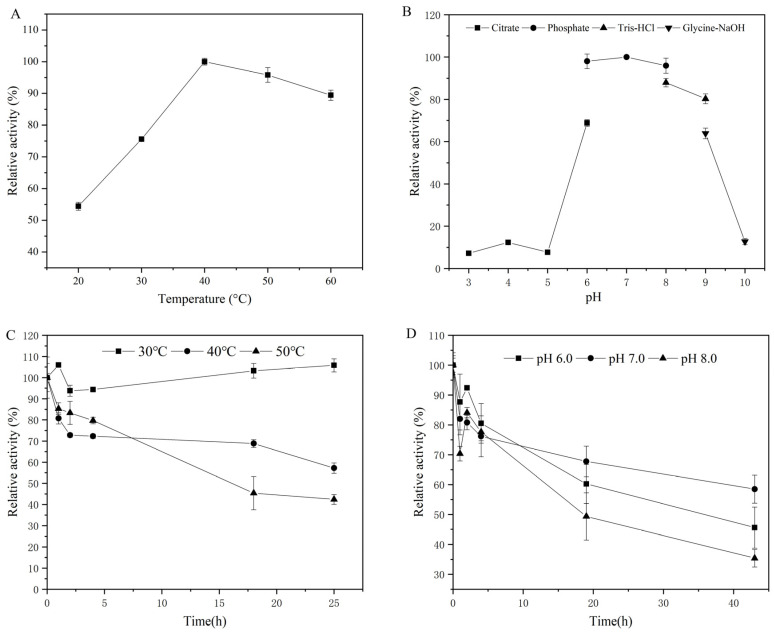
Biochemical characteristics of the purified GAO-5F. (**A**) Optimum temperature. (**B**) Optimum pH. (**C**) Thermal stability of GAO-5F at 30, 40, and 50 °C. (**D**) pH stability of GAO-5F at pH 6.0, 7.0, and 8.0.

The oxidative activity of purified GAO-5F was characterized using D-galactose as the substrate. The optimum reaction temperature of purified GAO-5F was at 40 °C, and it exhibited about 90% of the maximal activity at 60 °C (Figure 4A). The optimal galactose oxidase activity of GAO-5F occurred at pH 7.0 in a 20 mM phosphate buffer, and it showed more than 80% of the maximal activity at pH 6.0–9.0 (Figure 4B). This result is similar to other galactose oxidases. For example, the optimal activities of galactose oxidases from *F. subglutinans*, *F. sambucinum*, *F. oxysporum,* and *F. acuminatum* were observed at 30, 35, 40, and 30 °C, and pH values of 7.0, 6.0–7.5, 7.0, and 7.0, respectively [32,33,34,40]. The thermal stability of GAO-5F decreased with increasing temperature. No significant decrease in enzyme activity was observed when GAO-5F was stored at 30 °C for 25 h. Up to 40% of the enzyme activity was retained when GAO-5F was stored at 50 °C for 25 h (Figure 4C). The measured thermostability is superior to those galactose oxidases reported from other *Fusarium* strains. For example, the galactose oxidase from *F*. *oxysporum* lost activity at less than 5 h of incubation at 50 °C [33]. The relative activity of galactose oxidase from *F*. *subglutinans* decreased to about 20% after 10 min incubation at 50 °C [34]. The properties of high specific activity at high temperatures and appropriate thermostability may be beneficial to the modification of agarose, the gelling temperature of which was usually at 35–40 °C. GAO-5F showed the greatest stability at pH 7.0, with more than 60% activity retained after 40 h incubation (Figure 4D). However, there was a dramatic decrease in activity after incubation at pH 6.0 and 8.0, and they retained less than 50% activity after 40 h. This result was similar to the galactose oxidase from *F*. *subglutinans*, which retained 60% activity after 48 h of incubation [34].

Kinetic measurements were determined under standard reaction conditions using D-galactose as substrate. The *K*_m_, *V*_max_, *k*_cat_, and *k*_cat_/*K*_m_ of purified GAO-5F were determined to be 31.9 mM, 0.1636 µmol/min/mg, 129.1366 s^–1^, and 4.04 mM^–1^s^–1^, respectively. The affinity and catalytic efficiency of GAO-5F were moderate when compared with those values for reported galactose oxidases. The *K*_m_ of GAO-5F was higher than the galactose oxidase from *F. acuminatum* (16.2 mM) [40], whereas it was lower than those from *F. oxysporum* (47 mM) [32] and *F. sambucinum* (61 mM) [33]. The *k*_cat_/*K*_m_ of GAO-5F was higher than galactose oxidases from *F. sambucinum* (0.89 mM^–1^s^–1^) [33] and *F. oxysporum* (2 mM^–1^s^–1^) [32], but lower than that from *F. subglutinans* (92 mM^–1^s^–1^) [34].

The effects of different metal ions and compounds on the activity of GAO-5F were analyzed, and the results are shown in Table 2. Monovalent and divalent cations, such as Na^+^, K^+^, and Mg^2+^, inhibited enzyme activity to a minor extent, whereas Cu^2+^ enhanced enzyme activity by 1.42-fold. This observation is not unexpected because galactose oxidase is a copper-dependent enzyme [41]. As a chelator of metal ions, EDTA caused a clear decrease in the activity of GAO-5F, which indicated that the enzyme was a metalloenzyme and required Cu^2+^ as a cofactor to perform its oxidative function. This result is similar to published data for galactose oxidases from *F. sambucinum* [32]. CTAB had a significant inhibitory effect on the enzyme, which showed only 14% relative activity. Tween 80 was shown previously to improve the absorbance readings of the galactose oxidase reaction, and the results herein showed that the activity of GAO-5F increased in the presence of Tween 80 [40].

Galactose oxidase has broad substrate specificity along with strict regioselectivity, which is one of the most interesting characteristics of this enzyme. Galactose oxidase accepts a variety of primary alcohols, such as benzyl alcohol, glycerol, and oligosaccharides or polysaccharides (non-reducing end containing D-galactose). The activity of purified GAO-5F toward various substrates was determined and the results were shown in Table 3. It showed the highest activity with D-galactose and more than 95% relative activity with methyl-α-D-galactoside and D-(+)-raffinose. In addition, it also displayed approximately 60% relative activity toward D-(+)-melibiose and relatively low activities (about 4%) with lactose monohydrate. The results were similar to those of other *Fusarium* sources of galactose oxidases [33,40]. GAO-5F exhibited catalytic activity toward D-galactose but D-glucose, sucrose, and maltose were not its substrates, which verified the strict regioselectivity of the galactose oxidase. GAO-5F also showed activity against marine polysaccharides containing D-galactose, such as agarose and *κ*-carrageenan, which offers significant potential in modifying marine polysaccharides. However, it showed no activity toward other tested polysaccharides, including soluble starch, fructooligosaccharides, guar gum, and gum arabic. In addition, the activity toward agarose was relatively low and there is huge distance for improvement by protein engineering in the future.

### 3.4. Oxidation of Agarose by GAO-5F

**Figure 5:**
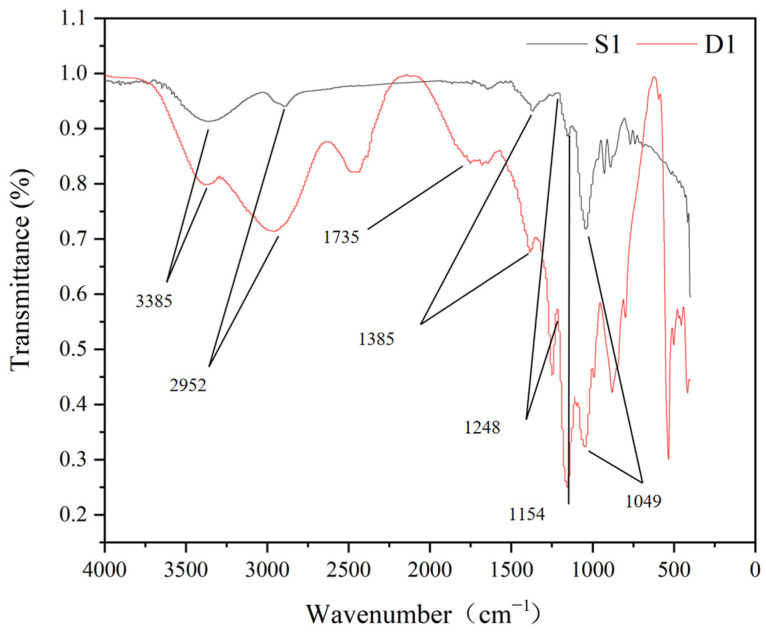
FTIR spectra of the substrate agarose (S1) and the polyaldehyde-based polymer (D1) oxidized by GAO-5F.

FTIR spectroscopy is used to examine molecular structures and chemical bonds, which enables the characterization and identification of compounds according to their unique structural features [42,43]. Infrared spectroscopy has been applied successfully to the structural analysis of agarose [44]. Figure 5 shows the FTIR spectra of the substrate agarose (S1) and the polyaldehyde-based polymer (D1) oxidized by GAO-5F. The FTIR spectrum of S1 is similar to the reported data [45], with the absorption peak at 3385.75 cm^–1^ representing the –OH bond stretching vibration, which is the characteristic peak of polysaccharides. The absorption peak at 2952 cm^–1^ represents the stretching vibration of the intra-ring C–H, whereas the peak at 1385 cm^–1^ represents the methyl group. The absorption peaks observed at 1248 cm^–1^, 1154 cm^–1^, and 1049 cm^–1^ indicate the presence of C–OH and C–O–C. The FTIR spectra of S1 and D1 were compared. In addition to the absorption peaks described above, D1 displayed a peak at 1735 cm^–1^, which corresponds to the characteristic absorption peak of an aldehyde group through the oxidation process and was not observed in the spectra of S1 [46]. Therefore, the absorption peak at 1735 cm^–1^ indicated the generation of polyaldehyde-based polymers in the D1 sample. This result proved that the galactose oxidase GAO-5F catalyzes the generation of a polyaldehyde-based polymer from agarose, which may be suitable for use as a crosslinking agent to form edible packaging films.

## 4. Conclusions

In this study, a galactose oxidase gene, *gao-5f*, from *F. odoratissimum* was cloned and heterologously expressed in *E. coli*. The biochemical properties of the purified recombinant GAO-5F were investigated in detail, revealing the highest activity toward D-galactose at pH 7.0 and 40 °C. In addition, GAO-5F displayed excellent thermal stability. Results from FTIR data showed that GAO-5F successfully catalyzed the oxidation of agarose to produce a polyaldehyde-based polymer. This result indicates the potential applicability of galactose oxidase in the field of edible packaging films. Combined with the strict regioselectivity, galactose oxidase has significant development prospects for potential industrial applications.

## Figures and Tables

**Table 1:** Purification of recombinant galactose oxidase.

Enzyme	Total Protein (mg)	Total Activity (U)	Specific Activity (U/mg)
GAO-5F	0.13	8.17	62.84 ± 0.021
GAO-13F	0.11	4.25	38.64 ± 0.023

**Table 2:** Effect of metal ions and chemicals on the activity of purified GAO-5F.

Compound	Concentration (mM)	Relative Activity (%) ^a^
CuCl_2_·2H_2_O	5	142 ± 3.59
Tween 80	0.25%	142 ± 1.58
None	-	100 ± 1.57
NaCl	5	87.9 ± 2.34
MgCl_2_·6H_2_O	5	85.1 ± 2.03
SDS	5	81.6 ± 1.98
KCl	5	80.5 ± 4.48
EDTA	5	76.7 ± 1.85
CTAB	5	14.8 ± 3.69

^a^ Activity expressed relative to the activity of GAO-5F without addition of any reagent.

**Table 3:** GAO-5F activity against different substrates.

Substrate	Concentration (mM)	Relative Activity (%)
D-Galactose	25	100 ± 3.57
Methyl-α-D-galactoside	25	99.7 ± 1.19
D-(+)-Raffinose	25	95.9 ± 2.93
D-(+)-Melibiose	25	57.6 ± 3.40
Agarose	0.1%	12.6 ± 1.04
*κ*-Carrageenan	0.1%	9.33 ± 0.52
Lactose monohydrate	25	4.56 ± 1.26
D-Glucose	25	0
Sucrose	25	0
Maltose	25	0
Glyoxal aqueous 40% solution	25	0
Ethylene glycol	25	0
D-(+)-Galacturonic acid monohydrate	25	0
Soluble starch	1%	0
Fructooligosaccharides	1%	0
Guar gum	1%	0
Gum arabic	1%	0

## Data Availability

The data are contained within the article.
